# Simulation and Experimental Study on Abrasive–Tool Interaction in Drag Finishing Edge Preparation

**DOI:** 10.3390/mi16101113

**Published:** 2025-09-29

**Authors:** Julong Yuan, Yuhong Yan, Youzhi Fu, Li Zhou, Xu Wang

**Affiliations:** 1 College of Mechanical Engineering, Zhejiang University of Technology, Hangzhou 310023, China; jlyuan@zjut.edu.cn (J.Y.); 15871572285@163.com (Y.Y.); 2 Key Laboratory of Special Purpose Equipment and Advanced Processing Technology, Ministry of Education and Zhejiang Province, Zhejiang University of Technology, Hangzhou 310023, China; 3 Institute of Interdisciplinary Research, Guangdong Polytechnic Normal University, Guangzhou 510665, China; yzfu@gpnu.edu.cn (Y.F.); zhouli@gpnu.edu.cn (L.Z.); 4 Heyuan-GPNU Institute, Guangdong Polytechnic Normal University, Heyuan 517500, China

**Keywords:** discrete element method, drag finishing, cutting edge preparation, process parameters, edge radius, edge shape factor *K*

## Abstract

Tool edge preparation is the process aimed at eliminating edge defects and optimizing the micro-geometric parameters of cutting tools. Drag finishing, the primary engineering method, subjects tools to planetary motion (simultaneous revolution and rotation) within abrasive media to remove burrs and micro-chips, thereby improving cutting performance and extending tool life. A discrete element method (DEM) model of drag finishing edge preparation was developed to investigate the effects of processing time, tool rotational speed, and rotation direction on abrasive-mediated tool wear behavior. The model was validated through milling cutter edge preparation experiments. Simulation results show that increasing the processing time causes fluctuating changes in average abrasive velocity and contact forces, while cumulative energy and tool wear increase progressively. Elevating tool rotational speed increases average abrasive velocity, contact forces, cumulative energy, and tool wear. Rotation direction significantly impacts tool wear: after 2 s of clockwise (CW) rotation, wear reached 1.45 × 10^−8^ mm; after 1 s of CW followed by 1 s of counterclockwise (CCW) rotation, wear was 1.25 × 10^−8^ mm; and after 2 s of CCW rotation, wear decreased to 1.02 × 10^−8^ mm. Experiments, designed based on simulation trends, confirm that edge radius increases with time and tool rotational speed. After 30 min of processing at 60, 90, and 120 rpm, average edge radius increased to 22.5 μm, 28 μm, and 30 μm, respectively. CW rotation increased the edge shape factor *K*, while CCW rotation decreased it. The close agreement between experimental and simulation results confirms the model’s effectiveness in predicting the impact of edge preparation parameters on tool geometry. Rotational speed control optimizes edge preparation efficiency, the predominant tangential cumulative energy reveals abrasive wear as the primary material removal mechanism, and rotation direction modulates the shape factor *K*, enabling symmetric edge preparation.

## 1. Introduction

In machining, tool performance directly impacts machining efficiency and workpiece quality [1]. Microscopic inspection after edge grinding often reveals geometric defects, such as micro-chips or serrations along the cutting edge [2]. These imperfections degrade workpiece quality and significantly reduce tool service life. Edge preparation eliminates these defects, reduces tool surface roughness, and diminishes residual stresses, thereby extending tool life [3].

Common edge preparation methods include Abrasive Jet Machining (AJM) [4], Brush Honing (BH) [5], and Drag Finishing (DF) [6]. Other methods include Magnetic Abrasive Finishing (MAF) [7], Abrasive Flow Machining (AFM) [8], Grinding (G) [9], and Electrochemical Machining (ECM) [10]. Drag finishing (DF) is a process in which the tool edge is fully immersed in abrasive media and subjected to planetary motion (simultaneous revolution and rotation), allowing the abrasives to remove material from the edge and form a desired cutting geometry. This method offers several advantages, including batch processing capability, low cost, and high efficiency. Planetary motion ensures uniform edge preparation across all cutting edges, which explains its widespread adoption not only for tool edge preparation but also for workpiece polishing [11].

Edge preparation techniques have been extensively studied. Wang et al. [12] prepared cemented carbide end mills using DF and rotary abrasive flow machining (RAFM). DF resulted in smaller edge radius changes with persistent defects and uneven edge profiles, while RAFM produced larger radius changes with effective defect removal and uniform rounding. Wang [13] compared three types of dispersed phases, measured their rheological properties, and proposed a new mechanism for shear-thickening behavior. This study demonstrated that dispersions containing phosphate functional groups offer better temperature resistance, making them suitable for prolonged polishing, while hydroxypropyl functional groups provide superior rheological properties for high-precision polishing. Guo [14] proposed an actively formed shear-thickening polishing (STP) method based on a numerical calculation model. After 30 min, the surface roughness of a bearing ring’s inner surface decreased from 131.27 nm to 27.14 nm and further reduced to 19.52 nm after 60 min, demonstrating the feasibility of the STP method for ultra-precision machining. Wang [15] investigated the effectiveness of shear-thickening polishing technology in preparing high-speed steel tap edges through simulation, analyzing the impact of workpiece speed, rotary speed, and inclination angle on pressure distribution and surface roughness during edge preparation. This work identified optimal edge preparation conditions under maximum pressure, improving edge consistency and minimizing roughness. Zhou [16] used discrete element simulations to compare and validate the influence of immersion depth, abrasive velocity, abrasive radius, and abrasive density on material removal rates for two materials. Results indicated that immersion depth significantly affects abrasive wear, while abrasive properties predominantly influence erosion wear. Peter [17] presented a cutting edge preparation method for cemented carbide milling tools using a prototype drag finishing system, demonstrating that spindle rotation speed is the dominant factor influencing edge radius, while feed rate has only a marginal effect. Dejin [18] studied the impact of different abrasive media on cutting edge surface roughness and revealed the material removal mechanisms and kinematics of drag finishing. Wang [19] compared three edge preparation methods—brushing, drag finishing, and wet abrasive jet machining—on carbide tool performance during orthogonal turning. Experiments confirmed that tools processed by drag finishing exhibited superior overall performance, including lower cutting temperatures, better stress distribution, reduced cutting forces, diminished flank wear, and lower machined surface roughness compared to other techniques. Czarniak [20] investigated the effect of drag finishing on tool life. Results showed that the positive impact on particleboard machining depended on carbide structure and hardness. A significant tool life extension (about 50%) was observed only with harder carbide (2240 HV10). No significant advantage was found for the softer carbide (1800 HV10). Yang [21] proposed an analytical model for WAJM-based cutting edge preparation that simulates the sharp-to-honed transition under varied process parameters. Validated on carbide and ceramic inserts, the model achieved a maximum edge-profile deviation ≤1 μm, mean errors <10% for *S*_γ_, *S*_α_ and *K*, and <15% for *D*_r_ and *D*_f_, and clarified how traverse speed and jet angle govern the resulting edge geometry. Wei [22] proposed a thermomechanical model for the initial flank-wear stage based on the tri-zone (sticking–transition–sliding) phenomenon; by coupling Waldorf’s slip-line fields with a calibrated 1-D thermal model and an Usui wear law, it resolves velocity, normal stress, and temperature along the tool–workpiece interface and predicts TiAlN-coated tool flank wear in Inconel 718 with ≤6.24% error and regional temperature deviations <3%, with SEM/EDS confirming adhesion-dominated wear and progressive coating degradation. Pérez-Salinas [23] investigated DF edge preparation (two grit sizes/three mixing ratios), varying immersion depth and drag time; an Repeatability and Reproducibility study and an ANN ranked ER drivers (depth > time > mix > grit), showed reproducibility comparable to brushing/blasting, and achieved approximately 94% ER-prediction accuracy, confirming DF’s feasibility for carbide tool edge preparation. Urbikain and de Lacalle [24] presented the first geometrical, time-domain model to predict surface topography in flank milling with circle-segment (oval-form) end mills, accounting for tool geometry, feed rate, radial immersion, runout, and 5-axis orientation; validation on Al7075-T milled walls matched experiments, providing a practical basis for optimizing reliable machining of complex aerospace components. Uriarte [25] developed a modified mechanistic model for predicting cutting forces in the micromilling of hardened tool steel (H13, 60 HRC). Their model, adapted from the conventional end-milling force model and validated using micro end mills with diameters as small as 0.1 mm, demonstrated consistent agreement between simulated and measured forces, providing a basis for estimating tool deflection and actual tool-path. Del Olmo [26] proposed an efficient monitoring method for the broaching of mission-critical firtree slots by integrating real-time sensor data (accelerometers, load cells, and motor drive consumption) with offline tool wear inspection. Their experimental results demonstrated the sensitivity of process signals to tool degradation, establishing a practical approach for daily production to prevent costly workpiece scrap and tool failure. Although these studies analyzed force characteristics, edge radius, and edge surface roughness, providing valuable process optimization references, they did not fully elucidate material removal mechanisms or investigate the edge shape factor.

Driven by advances in numerical computation and contact mechanics, the discrete element method (DEM) provides a vital approach for analyzing interactions between discrete particles and geometries [27].

This study establishes a DEM model of drag finishing edge preparation to analyze the effects of time, tool rotational speed, and rotation direction on the motion of abrasives, contact forces, cumulative energy, and tool wear. Concurrently, drag finishing experiments on milling cutters were conducted to analyze variations in edge radius with time and tool rotational speed, and the variation in shape factor *K* with rotation direction. Experimental results validate the simulation model’s reliability.

## 2. Establishment of the Tool Edge Preparation Simulation Model

### 2.1. Tool Edge Preparation Process

**Figure 1:**
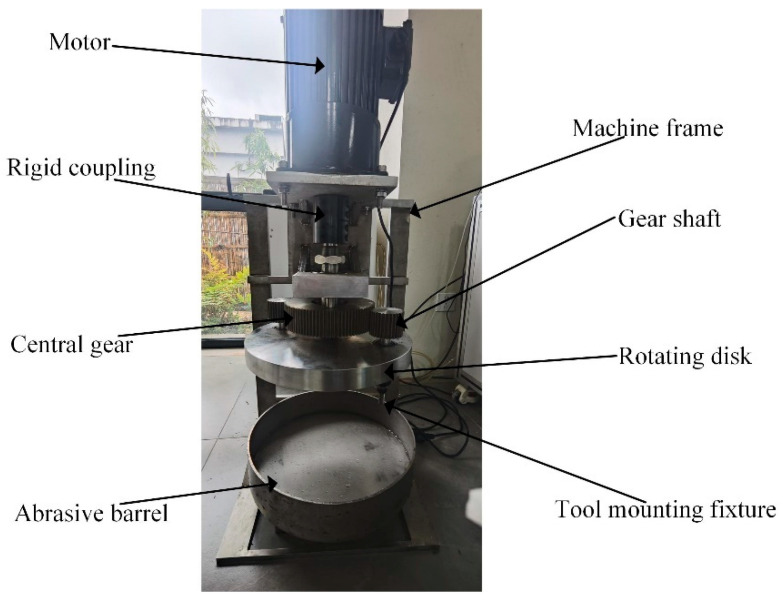
The drag finishing machine.

Edge preparation was performed using a drag finishing machine (Figure 1). Key components include a motor, rigid coupling, rotating disk, central gear, gear shafts, abrasive barrel, machine frame, and tool mounting fixture. This single-stage epicyclic system generates planetary motion through a simple mechanical device. A set of tools is mounted on the fixture, where the motor drives the rotating disk via the rigid coupling, imparting orbital motion to the gear shafts and tools. Simultaneously, meshing between the gear shafts and central gear drives tool rotation, establishing compound planetary motion (simultaneous revolution and rotation). The abrasive medium—a mixture of walnut shell grit, brown fused alumina, and silicon carbide in a specified ratio—fills the barrel. Fully immersed tools undergo planetary motion within this medium, enabling omnidirectional edge preparation. Continuous abrasive-edge contact and collisions remove micro-defects, achieving efficient and uniform edge micro-geometry. While tools follow deterministic kinematic paths derived from planetary mechanics, abrasive motion emerges stochastically from tool interactions, resulting in a highly complex process [28].

**Figure 2:**
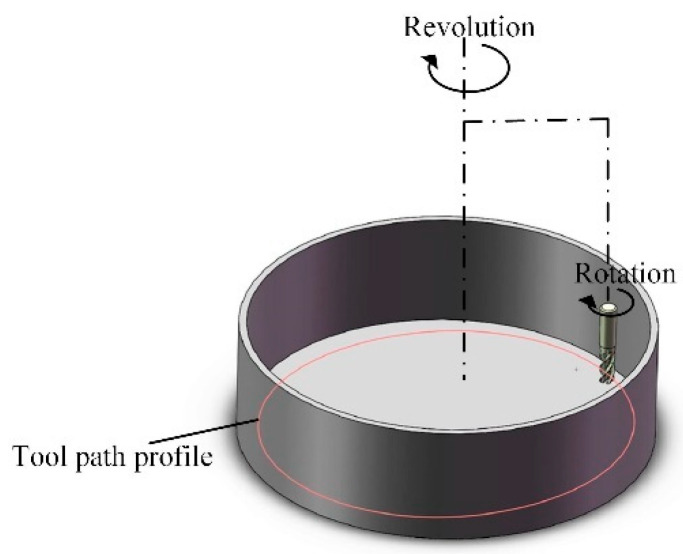
The schematic of the cutting toolpath.

Figure 2 illustrates the schematic of the cutting toolpath, depicting the trajectory of single-stage planetary motion (simultaneous revolution and rotation).

### 2.2. Establishment of the Simulation Models

**Figure 3:**
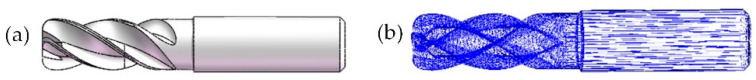
Milling cutter model: (**a**) three-dimensional model; (**b**) discretized model.

Figure 3a shows the three-dimensional model of the milling cutter, with the following key dimensions: a cutting diameter of 10 mm, a cutting length of 40 mm, a shank diameter of 10 mm, and a shank length of 30 mm. After importing the model into EDEM and meshing it (Figure 3b), the mesh size is 1.05 mm. The discretized model consisted of 18,066 element faces and 9035 nodes.

**Figure 4:**
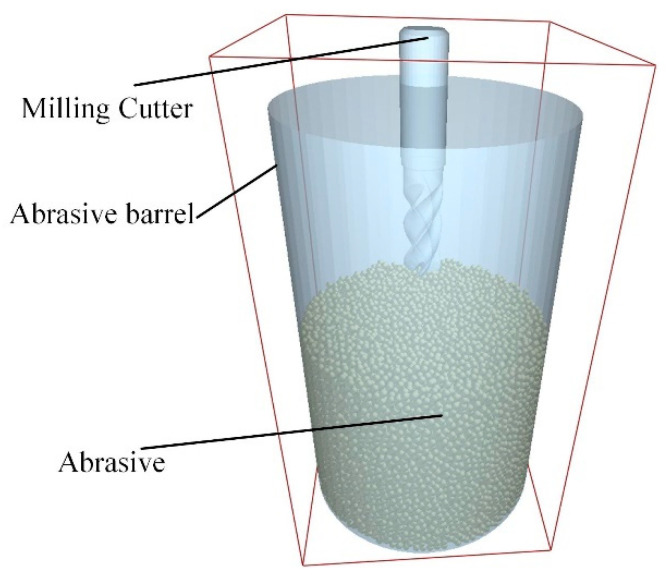
Simulation model.

Within the discrete element method (DEM) software EDEM software Altair EDEM 2022.0 (Professional Edition, Version 8.0.0), an abrasive barrel was modeled with stainless steel material properties. A particle generator was created using a single-sphere model with a defined radius to reduce computational complexity. It should be noted that the spherical particle model represents a simplification, as real 20-mesh SiC grit is angular and irregular. These geometric characteristics result in sharper contact points, elevated local stresses, and distinct rolling/sliding behavior compared to spheres. Consequently, the current model may underestimate localized stress concentrations and associated wear rates. Particles were generated via the Static Factory method (Fill Section) with an initial velocity of −0.2 m/s along the *Z*-axis, a value selected to replicate practical loading conditions and facilitate efficient packing. After generation (completed in 2 × 10^−5^ s, yielding 29,909 particles), the system was equilibrated under gravity for 0.5 s. This duration ensured velocities decayed to a negligible level (max: 3 × 10^−5^ m/s), establishing a mechanically stable initial bed representative of the real machine’s pre-start condition before subsequent simulation phases. This state could eliminate initial velocity effects of the abrasive particles on simulation results. The final simulation model is shown in Figure 4.

### 2.3. Material Contact Parameters and Contact Model

Contact parameters and material properties were assigned based on the GEGM database in EDEM. Silicon carbide was designated as the abrasive material, with its corresponding contact parameters against the milling cutter summarized in Table 1. The DEM solver uses explicit time integration, so the time step must be shorter than the fastest elastic/contact response in the system. The controlling scale is set by the smallest, stiffest abrasive grains. Based on the SiC properties and the smallest grain size employed, a conservative time step of 6.6578 × 10^−7^ s is selected. This choice ensures that short duration contacts are resolved by many sub-steps, keeping particle overlaps small.

For particle-particle contact models, the Hertz–Mindlin (no slip) model and the Standard Rolling Friction model were selected. For particle-tool geometry contact models, the Hertz–Mindlin (no slip) model, Standard Rolling Friction model, Archard Wear model, and Relative Wear model were implemented [29].

The Hertz–Mindlin (no slip) model provides an accurate and efficient method for force calculation, making it suitable for simulating physical behaviors such as particle collisions, friction, and deformation. The Standard Rolling Friction model accounts for rolling resistance by applying a torque to contacting surfaces. The Archard Wear model is based on the idea that the volume of material removed from the surface is proportional to the frictional work carried out by particles moving over it. The Relative Wear model identifies regions of high impact and abrasive wear on the equipment within a simulation. It provides four relative wear properties: Normal Cumulative Energy, Tangential Cumulative Energy, Normal Cumulative Force, and Tangential Cumulative Force.

## 3. Simulation Research and Analysis

**Figure 5:**
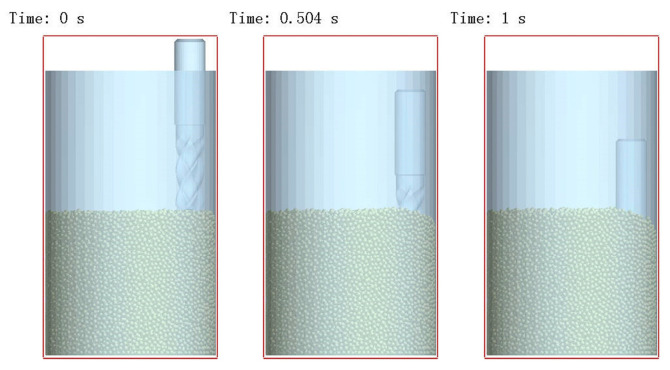
Tool descent process.

Simulation parameters were set to replicate actual operational conditions. Figure 5 illustrates the tool descent process: the tool descended uniformly at 3.5 × 10^−2^ m/s, reached the target immersion depth after 1 s, and halted. During descent, contact with silicon carbide particles generated impact collisions. As a result, at 1 s, the average abrasive velocity was 2.71 × 10^−3^ m/s, the contact force was 0.838 N, and the tool wear was 9.05 × 10^−10^ mm. This state was saved as the initial condition to eliminate the influence of the tool descent on subsequent simulation phases. Afterward, tool revolution and rotation were activated, with the rotational speed set to three times the revolution speed. EDEM employs the dynamic relaxation method to solve equations. This method requires the computation of particle/geometry forces, velocities, and positions at each timestep based on prior states. As a result, the computational load is substantial. Therefore, considering factors such as simulation efficiency, the simulation duration should be reasonably set.

### 3.1. Effects of Time and Tool Rotational Speed on the Edge Preparation Process

Simulation conditions included 20-mesh silicon carbide (SiC) abrasives, clockwise tool rotation (viewed top-down), rotation speeds of 90, 120, 150, and 180 rpm, with corresponding revolution speeds of 30, 40, 50, and 60 rpm, and a total duration of 2 s.

#### 3.1.1. Effects of Time and Tool Rotational Speed on the Motion of the Abrasive

**Figure 6:**
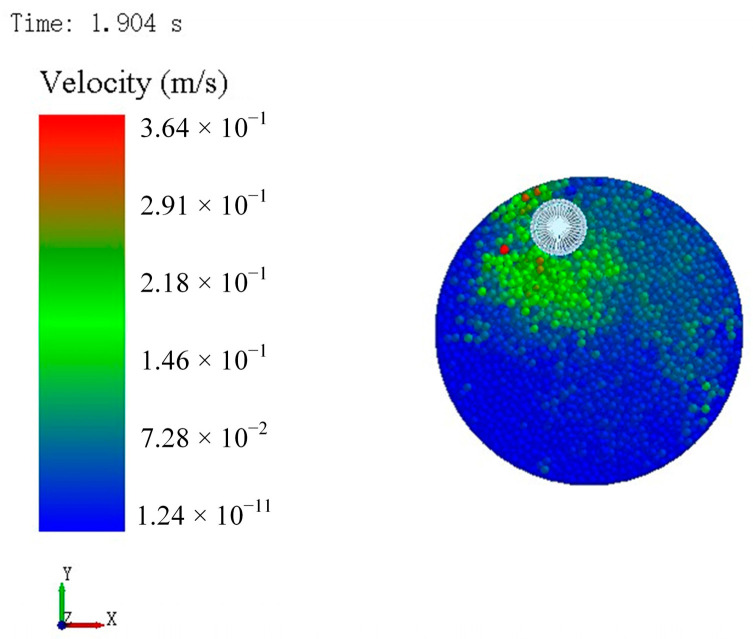
The velocity distribution of abrasive particles.

**Figure 7:**
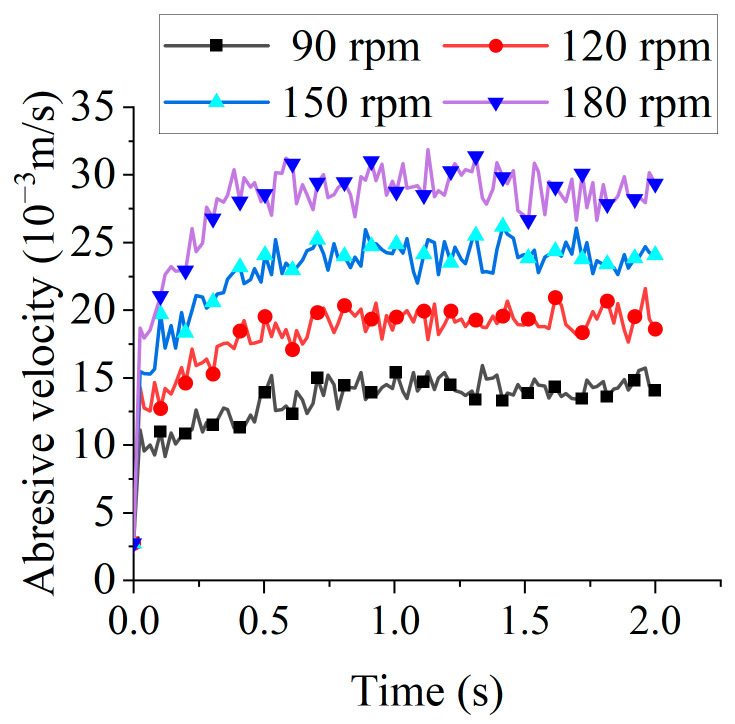
The average velocity of abrasive particles under different tool rotational speeds.

Higher abrasive kinetic energy increases contact frequency with the tool edge, thereby enhancing edge preparation efficiency. Figure 6 displays the velocity distribution of abrasive particles at 90 rpm and at 1.904 s. Figure 7 presents the average velocity of abrasive particles under different tool rotational speeds. Due to the stochastic nature of collisions, the average abrasive velocity fluctuated over time. The time-averaged velocity of the abrasive media over the 2-s interval demonstrated a pronounced increasing trend with the rotational speed of the tool, rising from 0.0134 m/s at 90 rpm to 0.0279 m/s at 180 rpm. This corresponds to an enhancement of more than 100%, indicating that elevated rotational speeds impart substantially greater kinetic energy to the abrasive particles.

#### 3.1.2. Effects of Time and Tool Rotational Speed on Contact Forces

**Figure 8:**
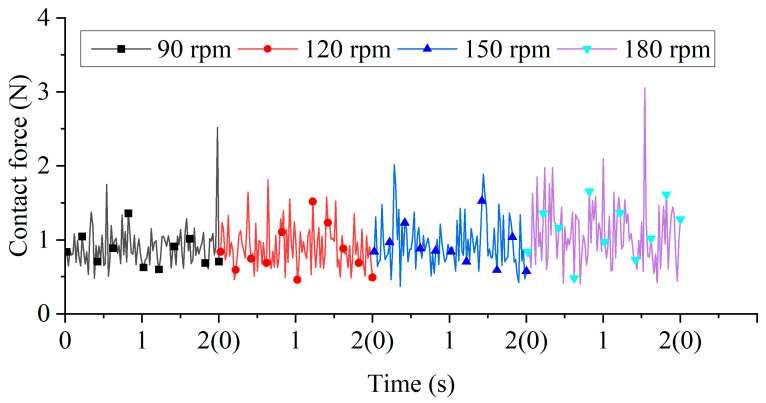
Contact forces under different tool rotational speeds.

Contact forces correlate with material removal rates and typically enhance preparation efficiency. Figure 8 illustrates contact forces under different tool rotational speeds. Temporal fluctuations in force magnitudes arose from collision stochasticity, with force spikes occurring during particle collisions in the tool shank region. Coarse meshing in this area (compared to the finer meshing at the edge) created a larger equivalent radius. Since normal and tangential forces scale with equivalent radius, these collisions generated transient force peaks. Processing raw data with a mean function yielded the following average contact forces over the 0–2 s interval: 0.905 N (90 rpm), 0.912 N (120 rpm), 0.948 N (150 rpm), and 1.081 N (180 rpm). The average contact forces on the tool increased gradually but modestly with tool rotational speed, primarily due to the simulation’s small revolution radius. A larger revolution radius would increase the tool’s linear velocity, resulting in proportionally greater contact forces.

#### 3.1.3. Effects of Time and Tool Rotational Speed on Cumulative Energy of the Tool

**Figure 9:**
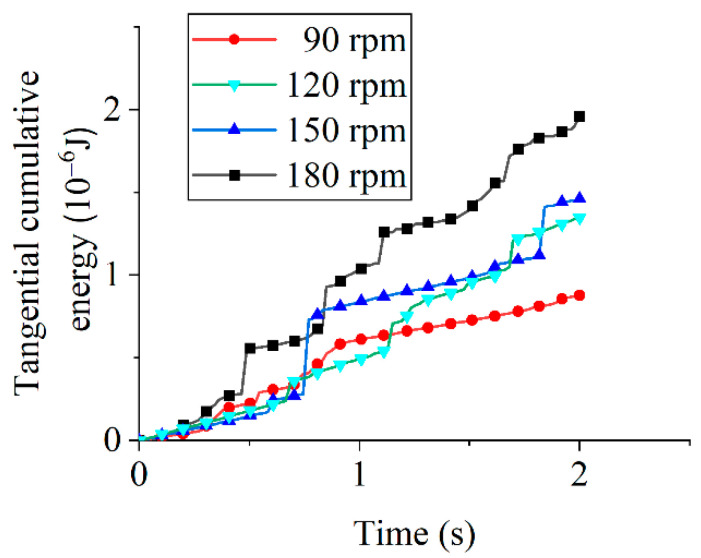
Tangential cumulative energy under different tool rotational speeds.

Figure 9 displays tangential cumulative energy under different tool rotational speeds. The tangential cumulative energy accumulated over time and increased substantially with tool rotational speed. By the end of the 2-s simulation, the energy at 180 rpm was approximately 2.2 times greater than that at 90 rpm.

**Figure 10:**
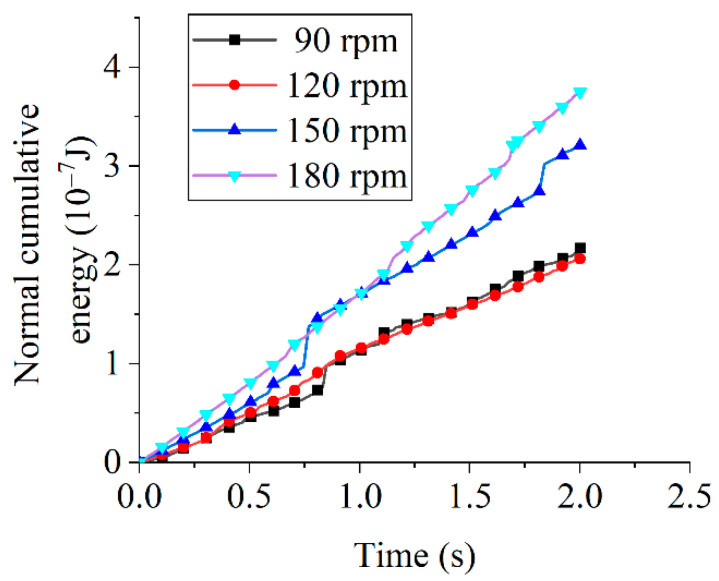
Normal cumulative energy at different tool rotational speeds.

Figure 10 presents normal cumulative energy at different tool rotational speeds. At 2 s, measured energies were 2.17 × 10^−7^ J (90 rpm), 2.06 × 10^−7^ J (120 rpm), 3.21 × 10^−7^ J (150 rpm), and 3.75 × 10^−7^ J (180 rpm).Although the general trend indicates an increase in normal cumulative energy with time and tool rotational speed, the observed lower value at 120 rpm relative to 90 rpm can be attributed to the limited simulation duration, which results in insignificant differentiation in cumulative energy values.

Comparative analysis of tangential and normal cumulative energies (Figure 9 and Figure 10) reveals substantially greater tangential energy growth across all tool rotational speeds, with normal energy attributed to erosion wear and tangential energy to abrasive wear. The higher tangential energy growth rate indicates that shear-driven abrasive wear dominates edge removal, while the lower normal energy reflects the minor contribution of impact-type erosion. In drag finishing, abrasive particles maintain sustained sliding and rolling contact with the tool edge: normal energy primarily serves to establish contact pressure and occasional impact (resulting in limited erosive chipping), whereas tangential energy supplies the frictional work driving micro-cutting and plowing of surface asperities, which continuously detaches material as fine debris. Thus, abrasive wear constitutes the dominant material removal mechanism regardless of rotational speed, and given the established proportionality between cumulative energy and tool wear [30], wear evolution patterns can be quantitatively deduced.

#### 3.1.4. Effects of Time and Tool Rotational Speed on Tool Wear

**Figure 11:**
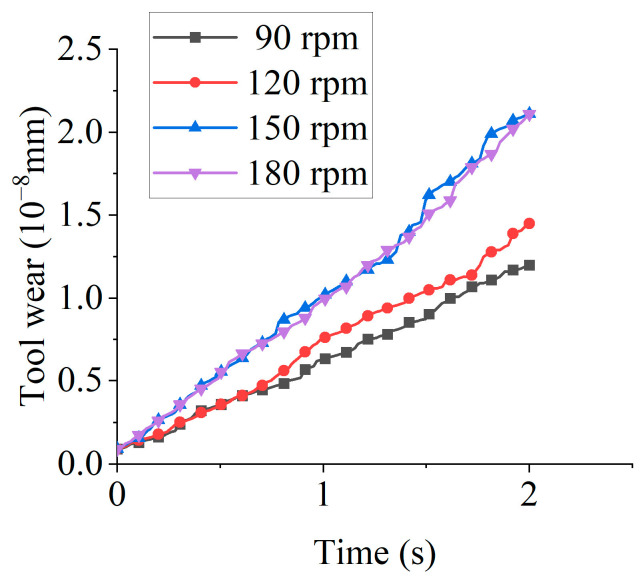
The tool wear under different tool rotational speeds.

Figure 11 illustrates the tool wear under different tool rotational speeds. Tool wear increased linearly with time, as demonstrated by the linear regression at 90 rpm: *y* = 2 × 10^−9^·*t* + 6 × 10^−10^, where *y* denotes the tool wear volume (mm) and *t* represents time (s), with *R*^2^ = 0.9965, indicating that the model explains 99.65% of the variance. At 2 s, wear volumes measured 1.20 × 10^−8^ mm (90 rpm), 1.45 × 10^−8^ mm (120 rpm), 2.11 × 10^−8^ mm (150 rpm), and 2.11 × 10^−8^ mm (180 rpm). Wear increased with tool rotational speed from 90 to 150 rpm but plateaued at 180 rpm. This plateau arises because excessive rotational speeds reduce the effective particle dwell time, limiting the abrasive-surface interaction. Consequently, optimal speed selection maximizes edge preparation efficiency.

**Figure 12:**
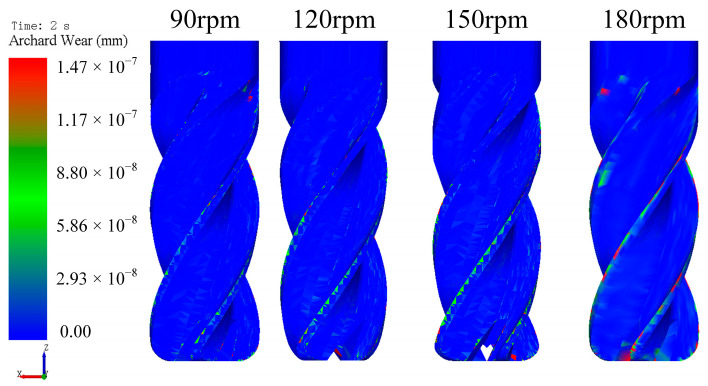
The contour plots of tool wear under different tool rotational speeds.

Figure 12 displays the contour plots of tool wear under different tool rotational speeds. Wear primarily concentrates at the cutting edge. Within the tested parameter range (90–150 rpm), wear magnitude increased proportionally with tool rotational speed.

### 3.2. Effect of Rotation Direction on Abrasive Particle Motion State

Simulation conditions included 20-mesh silicon carbide (SiC) abrasives, a tool rotational speed of 120 rpm, a corresponding revolution speed of 40 rpm, and a total duration of 2 s. The setting of tool simulation conditions is detailed in Table 2.

#### 3.2.1. Effects of Rotation Direction on the Motion of the Abrasive

**Figure 13:**
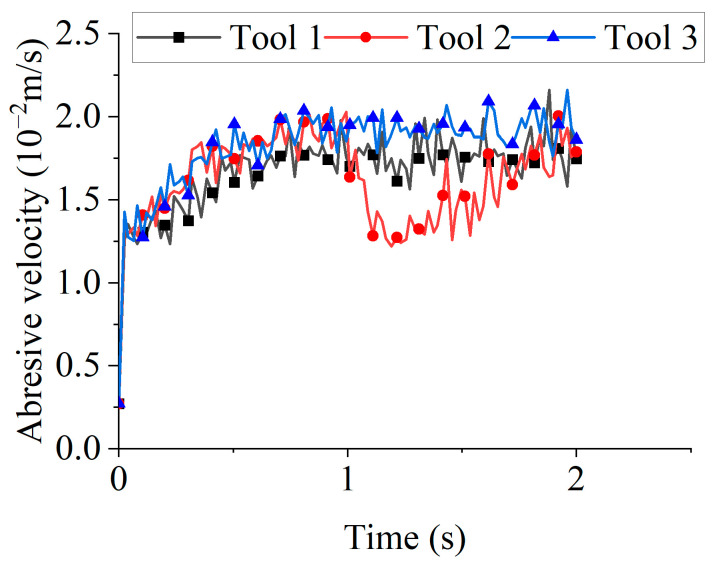
The average abrasive velocities for different rotation directions.

Figure 13 displays the average abrasive velocities for different rotation directions. During the 0–1 s interval, Tools 2 and 3 exhibited higher velocities than Tool 1, which can be attributed to geometric differences. Under clockwise rotation, a greater number of abrasive particles experienced impact collisions with the tool, transferring more kinetic energy to the particles. Consequently, clockwise rotation yielded higher average velocities than counterclockwise rotation during this interval.

**Figure 14:**
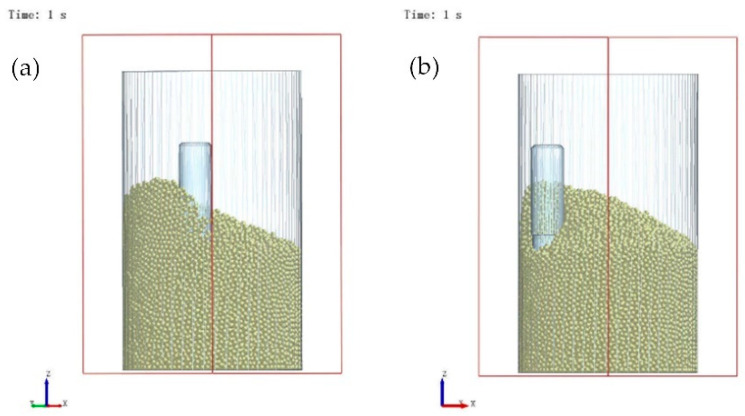
State of Tool 2 and the abrasive particles at 1 s: (**a**) Perspective 1 (perpendicular to the *Z*-axis, with an *X*-axis angle of 135°); (**b**) Perspective 2 (perpendicular to the *Z*-axis, with an *X*-axis angle of 45°).

Figure 14 shows the state of Tool 2 and the abrasive particles at 1 s. Because abrasive particles had accumulated at the clockwise-rotating end during the previous second, the reversal to counterclockwise rotation at 1 s produced far fewer particle–tool contacts than under the initial or steady-state rotation. This drastic reduction in the number of particles available for collision and wear led to a sharp drop in the average particle velocity. The velocity then gradually recovered as the number of contacting particles increased with prolonged counterclockwise rotation, eventually aligning closely with that of Tool 1.

#### 3.2.2. Effects of Rotation Direction on Contact Forces

**Figure 15:**
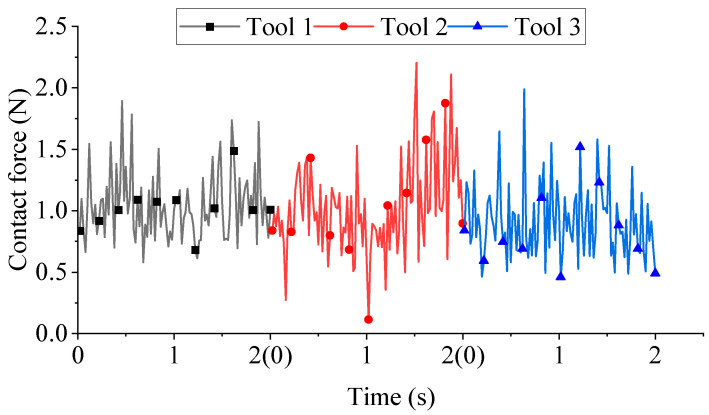
Contact forces for different rotation directions.

Figure 15 depicts the contact forces for different rotation directions. At *t* = 1 s, when Tool 2 transitions from forward to reverse rotation, the state illustrated in Figure 14 emerges. Following the reversal of rotation direction, the number of particles in contact with the cutter decreases sharply. Consequently, a rapid reduction in contact forces occurs immediately after 1 s, followed by a gradual increase over time. Mean forces computed over the 0–2 s interval using a mean function yielded 1.041 N (Tool 1), 1.021 N (Tool 2), and 0.912 N (Tool 3). Force magnitude exhibited limited sensitivity to rotation direction.

#### 3.2.3. Effects of Rotation Direction on Cumulative Energy of the Tool

**Figure 16:**
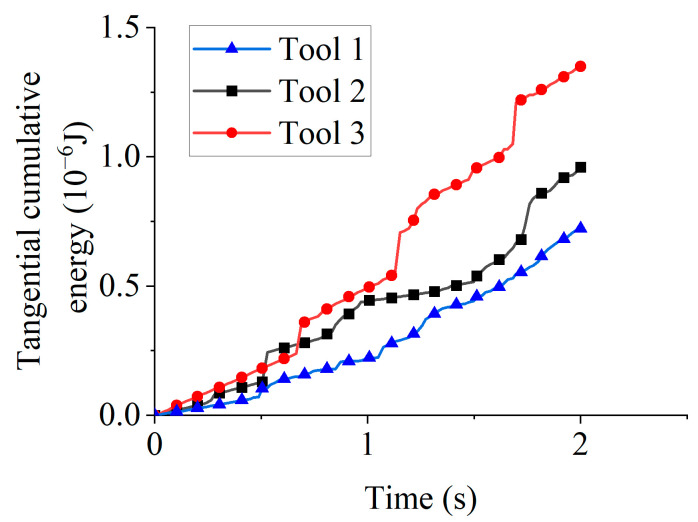
Tangential cumulative energy for different rotation directions.

Figure 16 displays tangential cumulative energy for different rotation directions. At 1 s, the tangential cumulative energy measured 2.19 × 10^−7^ J (Tool 1), 4.39 × 10^−7^ J (Tool 2), and 4.9 × 10^−7^ J (Tool 3). Enhanced particle-tool contact during clockwise rotation enabled Tools 2 and 3 to accumulate more tangential energy than Tool 1 during the 0–1 s interval. At 2 s, the tangential cumulative energy reached 7.22 × 10^−7^ J (Tool 1), 9.6 × 10^−7^ J (Tool 2), and 1.35 × 10^−6^ J (Tool 3). The lower tangential cumulative energy of Tool 2 relative to Tool 3 resulted from initiating counterclockwise rotation at 1 s.

**Figure 17:**
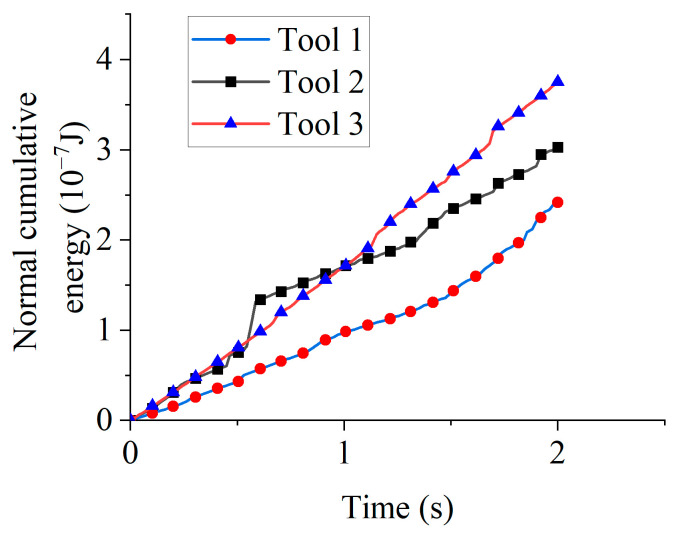
Normal cumulative energy for different rotation directions.

Figure 17 illustrates normal cumulative energy for different rotation directions. At 1 s, the normal cumulative energy values were 9.71 × 10^−8^ J (Tool 1), 1.69 × 10^−7^ J (Tool 2), and 1.70 × 10^−7^ J (Tool 3). Increased particle engagement during clockwise rotation enabled Tools 2 and 3 to accumulate higher normal energy than Tool 1 during the 0–1 s interval. At 2 s, the normal cumulative energy values were 2.42 × 10^−7^ J (Tool 1), 3.03 × 10^−7^ J (Tool 2), and 3.75 × 10^−7^ J (Tool 3). Tool 2’s reduced cumulative energy relative to Tool 3 similarly originated from counterclockwise rotation initiation at 1 s.

Comparative analysis of tangential and normal cumulative energies for identical tools (Figure 16 and Figure 17) reveals substantially greater tangential energy accumulation across all rotation directions. Therefore, abrasive wear dominates material removal in drag finishing edge preparation, regardless of rotation direction.

#### 3.2.4. Effects of Rotation Direction on Tool Wear

**Figure 18:**
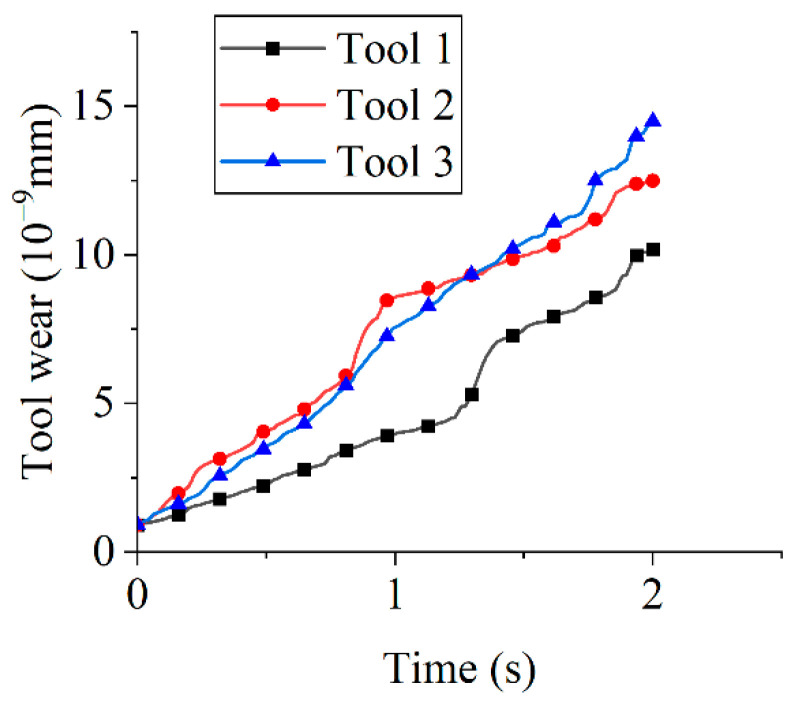
Tool wear for different rotation directions.

**Figure 19:**
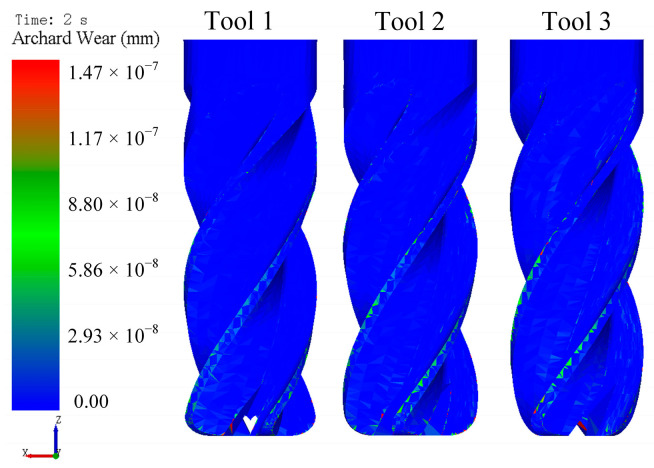
Contour plots of tool wear under different rotation directions.

Figure 18 presents tool wear for different rotation directions. After the first second, clockwise rotation (Tools 2 and 3) resulted in more than double the wear compared to counterclockwise rotation (Tool 1), indicating higher wear under clockwise rotation than counterclockwise. The slight discrepancy in tool wear between Tool 2 and Tool 3 during the 0–1 s interval originated from time step settings in the simulation software. Smaller time steps enhance computational accuracy but require longer computational times. Elevated wear under clockwise rotation is attributable to greater particle engagement. At 2 s, the tool wear reached 1.02 × 10^−8^ mm (Tool 1), 1.25 × 10^−8^ mm (Tool 2), and 1.45 × 10^−8^ mm (Tool 3), demonstrating that the rotation direction significantly influences material removal effectiveness across edge faces. During the 1–2 s interval, direction reversal in Tool 2 yielded wear rates consistent with counterclockwise rotation, resulting in lower wear compared to sustained clockwise rotation (Tool 3). Contour plots of tool wear under different rotation directions are presented in Figure 19.

## 4. Edge Preparation Experiments and Results

### 4.1. Experimental Design

The experimental program was rigorously designed to establish a direct, quantitative correspondence with the DEM simulation outcomes. The validation methodology was based on aligning key experimental measurements with specific predictions from the simulation model. By aligning experimental parameters with these numerical insights, the validation tests directly probe the predicted dependencies of edge radius and shape factor evolution. Specifically:

Time and Speed Dependence of Edge Radius: The simulation predicted that tool wear volume (a direct proxy for material removal and edge radius growth) increases approximately linearly with time and tool rotational speed within a certain range (Section 3.1.4, Figure 13). To validate this, experiments measured the edge radius evolution over time (10, 20, 30 min) at different rotational speeds (60, 90, 120 rpm).

Rotation Direction Dependence of Edge Geometry: The simulation revealed that rotation direction significantly affects the asymmetry of material removal on the rake and flank faces. To validate this, experiments measured the edge shape factor (K) after processing with different rotation directions (CW, CCW, CW to CCW).

**Figure 20:**
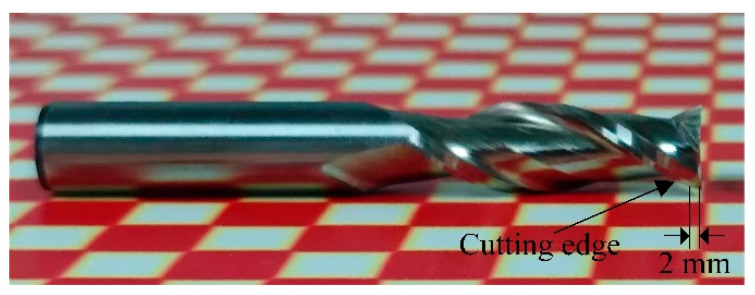
The two-flute keyway milling cutter.

The experiments were conducted using the drag finishing machine shown in Figure 1. The edge radius and shape factor *K* were measured using a 3D optical profiler (ZOLLER Inc., Ann Arbor, MI, USA). Measurements were taken 2 mm above the bottom cutting edge, as shown in Figure 20. Each of the cutter’s two flutes was measured twice, resulting in a mean value derived from four data points for each set of parameters. Edge morphology was characterized using a field-emission scanning electron microscope (SEM, ZEISS ∑IGMA, Oberkochen, Germany) and a laser scanning confocal microscope (KEYENCE VHX-S650E, Osaka, Japan). The two-flute keyway milling cutter (Figure 20) had a cutting diameter of 10 mm, a shank diameter of 10 mm, a cutting length of 30 mm, and an overall length of 70 mm. The cutter substrate was high speed steel without any coating. The abrasive medium consisted of walnut shell grit (average particle size 20 mesh), 20-mesh brown fused alumina, and 20-mesh silicon carbide in a 2:1:1 mass ratio. The SiC enables micro-cutting and edge rounding. The brown fused alumina refines surfaces, reducing the scratches caused by the SiC. The soft, fine walnut shell grit cushions, enhances flow, increases gentle contacts, and distributes tangential forces evenly.

### 4.2. Influence of Time and Tool Rotational Speed on Cutting Edge Radius

**Figure 21:**
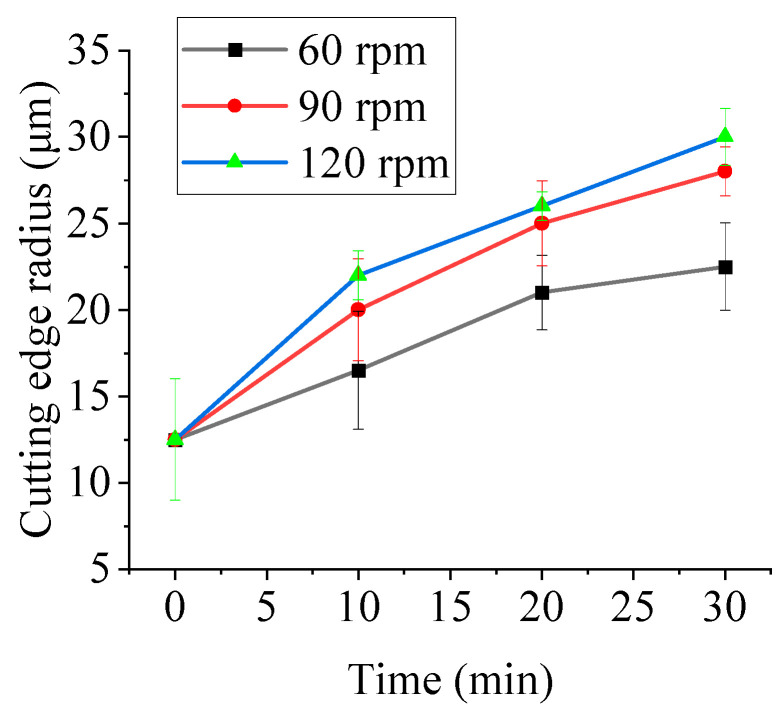
The variation in the edge radius under different tool rotational speeds.

Clockwise tool rotation was employed to investigate the effects of time and tool rotational speed on edge radius. Rotational speeds of 60, 90, and 120 rpm (with corresponding revolution speeds of 20, 30, and 40 rpm) were tested. The processing duration was 30 min, with edge radius measured at 10 min intervals. Figure 21 shows the variation in edge radius under different tool rotational speeds. The initial edge radius was 12.5 ± 3.5 μm. After 30 min of processing, the edge radius increased to 22.5 ± 2.5 μm (60 rpm), 28 ± 1.4 μm (90 rpm), and 30 ± 1.6 μm (120 rpm).

Figure 21 shows that, at a constant rotational speed, the edge radius increases over time in agreement with the simulated tool wear trend. At the highest speed, however, the growth rate slows and approaches a plateau. This saturation arises from two interacting effects. First, continuous clockwise rotation causes more frequent abrasive impacts on the rake face than on the flank face, so flank side material removal progressively lags behind that of the rake. As the rake edge becomes more rounded, the local contact angle decreases, reducing normal pressure and the cutting efficiency of individual particles. Second, higher rotational speed shortens the residence time of particles in contact with the edge, limiting the energy transferred per collision. Together these effects lower the incremental wear rate and lead to the observed saturation of edge radius growth.

**Figure 22:**
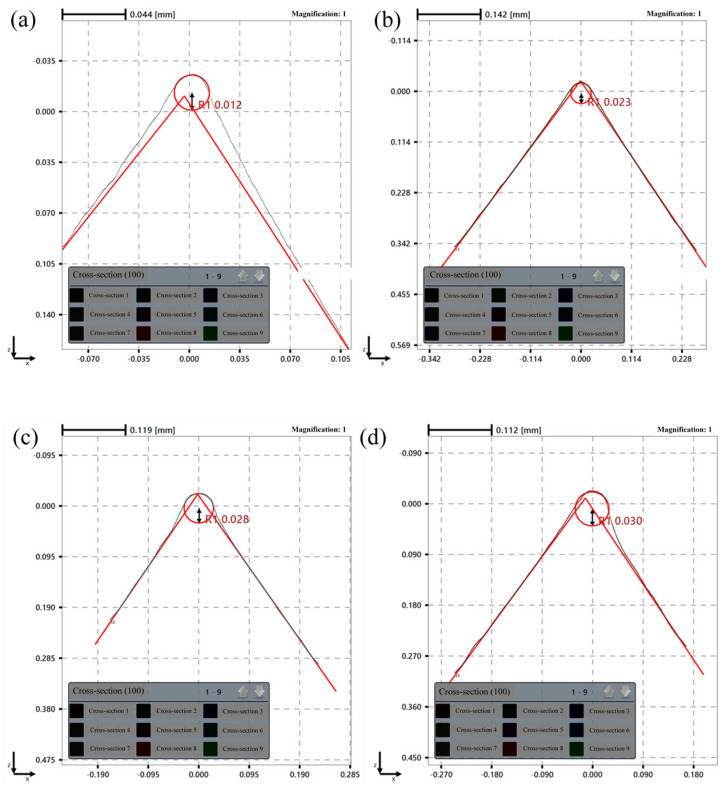
Edge radius results under different tool rotational speeds: (**a**) before preparation; (**b**) 60 rpm; (**c**) 90 rpm; (**d**) 120 rpm.

Simultaneously, under a fixed time condition, the edge radius increases with tool rotational speed, corroborating the simulation results, where tool wear similarly escalates with higher rotational speeds. Experimentally, this is attributed to the enhanced interaction forces between the tool and abrasive particles at elevated speeds, which promote more extensive edge material removal and consequent radius expansion. After 30 min of edge preparation, the measured edge radius results under different tool rotational speeds are presented in Figure 22.

**Figure 23:**
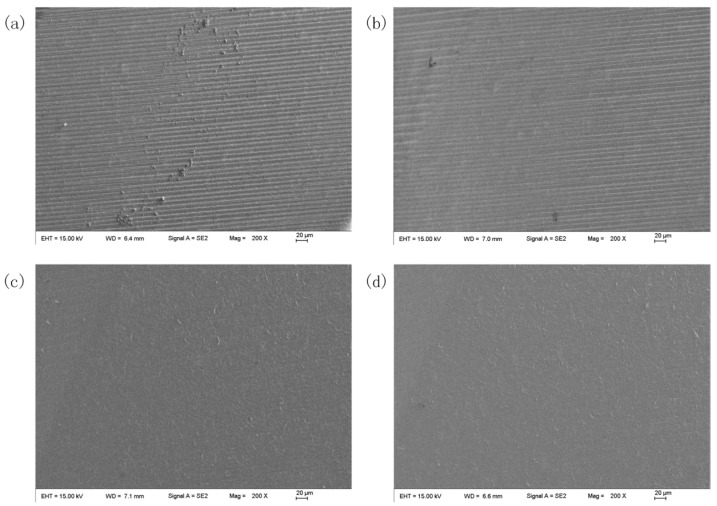
The tool edge surface morphology under different tool rotational speeds: (**a**) before edge preparation; (**b**) 60 rpm; (**c**) 90 rpm; (**d**) 120 rpm.

Figure 23 presents the tool edge surface morphology for different tool rotational speeds. Analysis via scanning electron microscopy (SEM) at 200× magnification reveals a substantial reduction in surface irregularities and grinding marks relative to unprepared edges. Progressive refinement of edge morphology is observed with increasing tool rotational speeds.

**Figure 24:**
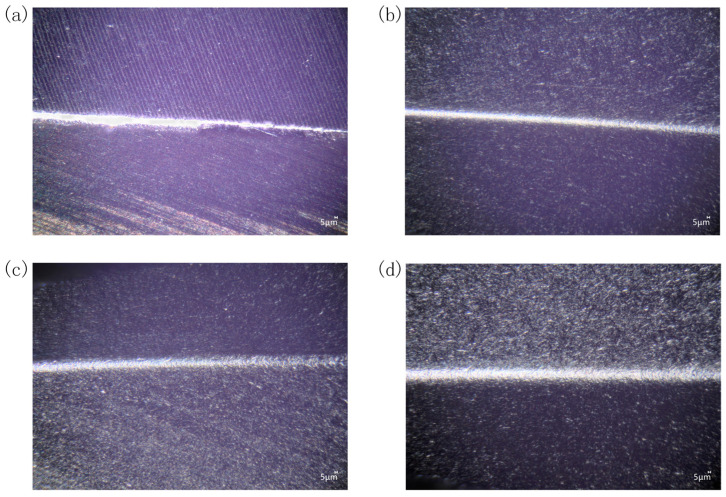
Cutting edge morphology for different tool rotational speeds: (**a**) before edge preparation; (**b**) 60 rpm; (**c**) 90 rpm; (**d**) 120 rpm.

Figure 24 presents cutting edge morphology for different tool rotational speeds. Observation under high depth-of-field microscopy (500× magnification) revealed enlarged, rounded, and uniform cutting edges after preparation, along with improved surface conditions.

### 4.3. Influence of Rotation Direction on Shape Factor K

The ideal tool tip is defined by the intersection of the tool’s rake face and flank face. The distances from this ideal tip to the rake face and flank face are denoted as *S*_γ_ and *S*_α_, respectively. The shape factor *K* is then defined by the equation [31]: *K* = *S*_γ_/*S*_α_.

The influence of rotation direction on *K* was investigated at a rotational speed of 90 rpm (with a revolution speed of 30 rpm) over a duration of 20 min. The experimental conditions for the cutting tools are detailed in Table 3. The value of *K* was measured at 10 min intervals during processing.

**Figure 25:**
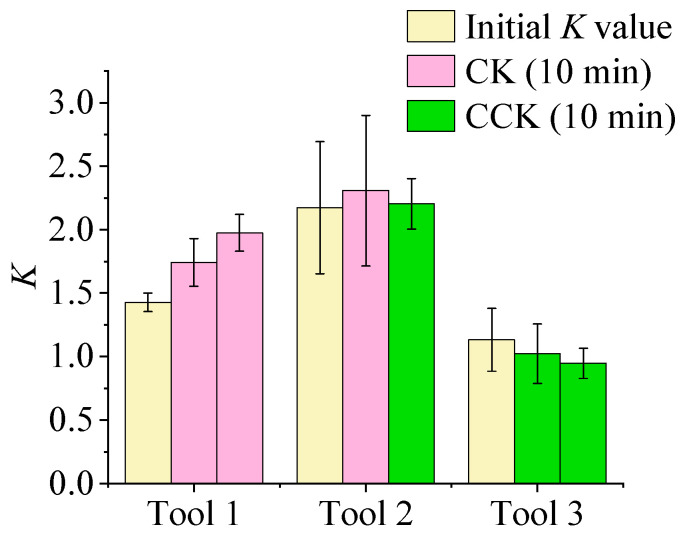
The variation in the edge shape factor *K* value for different rotation directions.

Figure 25 illustrates the variations in the edge shape factor *K* for different rotation directions. During the edge preparation process, clockwise rotation causes abrasive particles to primarily impact the rake face, thereby increasing *S*_γ_. Based on the definition *K* = *S*_γ_/*S*_α_, this increase results in a higher *K* value. In contrast, counterclockwise rotation directs most abrasive impacts toward the flank face, increasing *S*_α_ and consequently reducing *K*. Accordingly, Tool 1 (continuous clockwise rotation) exhibits a consistent increase in *K*; Tool 2 (clockwise followed by counterclockwise) demonstrates an initial rise followed by a decline; and Tool 3 (continuous counterclockwise) shows a steady decrease in *K*. These experimental outcomes align well with the simulation results, confirming that clockwise rotation leads to more substantial tool wear than counterclockwise rotation, and that the rotation direction dictates the material removal behavior on each tool face.

**Figure 26:**
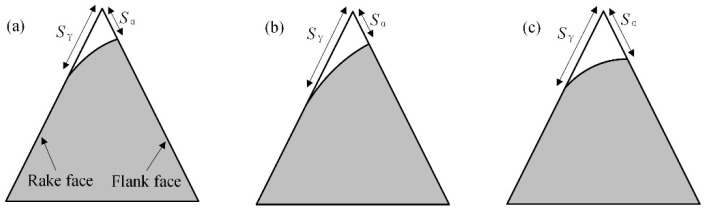
The variation in the shape factor *K* value of the cutting edge of the tool 2: (**a**) *K* = *S*_γ/_*S*_α_ = 2.17; (**b**) *K* = *S*_γ/_*S*_α_ = 2.30; (**c**) *K* = *S*_γ/_*S*_α_ = 2.20.

Taking the *K* value variation in Tool 2 as an example, Figure 26 clearly depicts its transition, characterized by an initial increase followed by a subsequent decrease. Therefore, the edge shape factor *K* can be precisely modulated by selecting an appropriate tool rotation direction.

By adjusting the drag finishing rotation direction, the edge shape factor *K* can be precisely tuned, directly linking edge geometry to cutting mechanics. As shown in Table 4 are the Cutting Performance Implications of Edge Shape Factor *K*. When *K* > 1, the rake side becomes more heavily rounded, enhancing edge strength and impact resistance but slightly diminishing sharpness. When *K* < 1, rounding occurs mainly on the flank side, maintaining a sharper rake edge and lowering cutting forces, though with reduced robustness. Consequently, selecting the appropriate rotation program—clockwise to increase *K* or counterclockwise to decrease it—allows the drag finishing process to tailor edge geometry to the functional demands of the subsequent machining operation, whether the goal is maximum tool toughness for hard or interrupted cuts or exceptional sharpness for fine finishing.

## 5. Conclusions and Prospects

### 5.1. Conclusions

This study established a discrete element model of drag finishing (DF) edge preparation using EDEM software Altair EDEM 2022.0 (Professional Edition, Version 8.0.0) to analyze the effects of time, tool rotational speed, and rotation direction on the motion of the abrasive, contact forces, cumulative energy, and tool wear. Key findings reveal the material removal mechanisms and provide process optimization guidelines, with experimental validation confirming model reliability:

1. Elevated tool rotational speeds intensify abrasive motion. Increasing the speed from 90 to 180 rpm raised the average abrasive velocity from 0.0134 m/s to 0.0279 m/s. Contact forces increase with the tool’s linear velocity. Edge preparation efficiency can be enhanced by increasing the revolution radius or tool rotational speed.
2. Tangential cumulative energy exhibits significantly higher growth rates than normal cumulative energy. After 2 s, tangential cumulative energy was 3–5 times greater than normal energy at equivalent tool rotational speeds, indicating that abrasive wear is the dominant material removal mechanism in tool edge preparation by DF. Tool wear increases approximately linearly with time. At 150 rpm, tool wear reached 2.11 × 10^−8^ mm. A further increase in tool rotational speed results in a plateau in wear volume. Thus, edge preparation efficiency can be maximized by rationally controlling the tool rotational speed.
3. Edge radius increases with both tool rotational speed and time: after 30 min of processing, the radius measured 22.5 μm (60 rpm), 28 μm (90 rpm), and 30 μm (120 rpm). Rotation direction modulates shape factor *K*: clockwise rotation increases *K* through preferential rake face removal, while counterclockwise rotation decreases *K* via flank face dominance. This enables precise geometric control for symmetric edge preparation.
4. The experimental results are consistent with the trends observed in the simulation. Edge radius increases with time at constant tool rotational speed and with tool rotational speed over fixed durations—consistent with simulated wear trends (tool wear increases with time and tool rotational speed). Similarly, *K* increases during clockwise rotation and decreases during counterclockwise rotation, consistent with the simulation findings (different rotation directions affect material removal effectiveness on different tool faces). These consistencies demonstrate the model’s predictive capability for parameter-dependent edge geometry evolution.

### 5.2. Prospects

To address the limitations identified and expand the study’s industrial applicability, future work will focus on three key directions. First, aiming at the ~2 s DEM simulation duration (far shorter than the tens of minutes of industrial drag finishing), a multi-scale modeling approach will be adopted: fine-grained simulation (retaining 20-mesh SiC particles and 1.05 mm cutter mesh) will capture initial contact mechanics and early wear, while coarse-grained modeling (merging fine abrasive particles into equivalent units) will extend the simulation to 30–60 min, enabling analysis of long-term effects like particle-packing evolution, heat accumulation, and steady-state wear.

Second, to reduce dependence on the current abrasive medium (walnut shell: brown fused alumina: SiC = 2:1:1), experiments with varied abrasive types (e.g., alumina-zirconia mixes) and particle sizes (10–40 mesh) will be conducted, exploring how abrasive properties affect edge radius growth and shape factor *K*, and updating the DEM model with new contact parameters to enhance its adaptability.

Finally, the model’s scope will be broadened to other tool geometries (e.g., taps) and substrate materials (e.g., high-speed steel), with long-duration industrial-scale trials (1–2 h) to validate simulation results under practical production conditions, ultimately establishing a more universal drag finishing edge preparation optimization framework.

## Figures and Tables

**Table 1:** Contact parameters.

	Abrasive (SiC)	Milling Cutter	Mechanistic Impact
Poisson’s ratio	0.14	0.3	Contact stress distribution.
Shear modulus (Pa)	1.796 × 10^11^	2.346 × 10^11^	Contact stiffness and collision force.
Density (kg/m3)	3.2 × 10^3^	1.27 × 10^4^	Particle inertia and impact energy.
Static friction coefficient	0.5	0.5	Sliding vs. plowing behavior.
Rolling friction coefficient	0.01	0.01	Particle rolling resistance.
Coefficient of Restitution	0.75	0.5	Collision rebound and energy loss.
Wear constant	2 × 10^−13^	2 × 10^−13^	Direct scaling of wear rate.

**Table 2:** The setting of tool simulation conditions.

	Tool 1	Tool 2	Tool 3
Rotation direction and duration	CCK for 2 s	CW for 1 s then CCW for 1 s	CK for 2 s

**Table 3:** The experimental conditions for cutting tools.

	Tool 1	Tool 2	Tool 3
Rotation direction and duration	CW for 20 min	CW for 20 min then CCW for 10 min	CCW for 20 min

**Table 4:** Cutting Performance Implications of Edge Shape Factor *K*.

Dimension	*K* > 1 (Greater Rake Face Rounding)	*K* < 1 (Greater Flank Face Rounding)
Geometry	Edge rounds toward rake face; local rake angle more negative	Edge rounds toward flank face; rake angle remains sharp
Stress distribution	Spreads tensile stress along rake, lowering notch stress and chipping risk	Concentrates stress at the lip, increasing micro-crack/chip risk
Cutting force and friction	Slightly higher cutting forces and frictional work	Lower cutting forces and friction
Minimum chip thickness	Larger; more prone to plowing at small feeds	Smaller; promotes early chip formation
Wear/chipping resistance	Stronger, more impact-resistant edge	Less robust edge; flank wear may accelerate
Typical applications	Hard/brittle materials, roughing, interrupted cuts	Ductile materials, fine finishing, low-feed machining

## Data Availability

Data are only available upon request due to restrictions regarding, e.g., privacy and ethics. The data presented in this study are available from the corresponding author upon request. The data are not publicly available due to their relation to other ongoing research.

## Abbreviations

CW	Clockwise
CCW	Counterclockwise
